# Sponge-like Scaffolds for Colorectal Cancer 3D Models: Substrate-Driven Difference in Micro-Tumors Morphology

**DOI:** 10.3390/biomimetics7020056

**Published:** 2022-05-05

**Authors:** Andrey Boroda, Yuliya Privar, Mariya Maiorova, Anna Skatova, Svetlana Bratskaya

**Affiliations:** 1 A.V. Zhirmunsky National Scientific Center of Marine Biology, Far Eastern Branch of Russian Academy of Sciences, 17, Palchevskogo Street, 690041 Vladivostok, Russia; borodandy@gmail.com (A.B.); maiorovamariya@gmail.com (M.M.); 2 Institute of Chemistry, Far Eastern Branch of Russian Academy of Sciences, 159, prosp.100-letiya Vladivostoka, 690022 Vladivostok, Russia; privar.juliya@gmail.com (Y.P.); militca2@mail.ru (A.S.)

**Keywords:** chitosan, carboxymethyl chitosan, cryogel, stiffness, 3D culturing

## Abstract

Macroporous scaffolds (cryogels) for the 3D cell culturing of colorectal cancer micro-tumors have been fabricated by cross-linking chitosan and carboxymethyl chitosan (CMC) with 1,4-butandiol diglycidyl ether (BDDGE) under subzero temperature. Due to the different intrinsic properties and reactivity of CMC and chitosan under the same cross-linking conditions, Young′s moduli and swelling of the permeable for HCT 116 cells cryogels varied in the broad range 3–41 kPa and 3500–6000%, respectively. We have demonstrated that the morphology of micro-tumors can be controlled via selection of the polymer for the scaffold fabrication. Although both types of the cryogels had low cytotoxicity and supported fast cell proliferation, round-shaped tightly packed HCT 116 spheroids with an average size of 104 ± 30 µm were formed in CMC cryogels (Young′s moduli 3–6 kPa), while epithelia-like continuous sheets with thickness up to 150 µm grew in chitosan cryogel (Young′s modulus 41 kPa). There was an explicit similarity between HCT 116 micro-tumor morphology in soft (CMC cryogel) or stiff (chitosan cryogel) and in ultra-low attachment or adhesive culture plates, respectively, but cryogels provided the better control of the micro-tumor’s size distribution and the possibility to perform long-term investigations of drug–response, cell–cell and cell–matrix interactions in vitro.

## 1. Introduction

The growth dynamics and complexity of tumors to a great extent are governed by spatiotemporal signaling, metabolic gradients, and mechanical characteristics of the in vivo tumor environment, which are notoriously difficult to mimic in vitro in biochemical and drug sensitivity tests in oncology [1,2]. Since the lack of cell–cell and cell–matrix interactions in routinely used 2D monolayer culture models was reported to result in a significant discrepancy between preclinical and clinical drug response [1,2,3], three-dimensional (3D) cell culturing became a very promising tool to mimic the physiological function of the extracellular matrix (ECM) and develop more adequate tumor models for studying hypoxia, cell aggregation, clustering, migration and proliferation [1]. 

Scaffold-dependent 3D cell cultivation is usually achieved by embedding cells into the hydrogels, whose composition, morphology and stiffness can be varied using different natural and synthetic polymers, and fabrication methods [3,4,5,6]. Substrate stiffness is known to significantly affect cell proliferation, migration, survival and invasiveness [7] that is especially important for the adherent cells, e.g., highly invasive colorectal cancer (CRC) [5,8,9] or breast cancer cells, which demonstrated faster cell migration, when confinement and substrate stiffness increased [10]. 

Protein-based hydrogels (collagen, Matrigel^®^, gelatin) are highly biocompatible and cell-adhesive, but, aside from the antigenicity potential due to the presence of animal-derived contaminants, they can provide only a limited range of elastic moduli (in average 0.1–4 kPa), while the stiffness of tissues with solid CRC tumors can be higher than 60 kPa [11,12]. Synthetic polymers offer a high tunability of chemical and mechanical properties of hydrogels, but in many cases have poor cellular responses [5] that can be overcome only by modification with cell adhesive substances, e.g., RGD peptide [3]. 

Polysaccharide-based (alginate, chitosan, pectin, hyaluronate, etc.) hydrogels are attractive alternatives to collagen and gelatin-based systems for growing cancer cell spheroids [2,5,13,14] due to a low toxicity, biocompatibility and wide spectrum of functional groups. The incubation of HCT 116 cells in alginate and alginate–gelatin hydrogels during the 8-day period yielded densely packed cell aggregates, whose growth was faster in alginate–gelatin hydrogel with a lower network density and higher porosity [2]. The addition of fibronectin to rather weak methylcellulose–hyaluronic hydrogel resulted in HCT 116 cells being more spread within the gel matrix at the first day of culturing, which was probably due to increased adhesiveness to the substrate; however, by the 7th day, only spheroids were observed in culture [15]. In addition to chemical differences in scaffolds or substrates, the stiffness can serve as a mechanical stimulus for cells. Inverse proportionality between the stiffness/hydrogel density and rate of spheroids growth was observed for HCT 116 cells culturing in thermo-responsive hybrid chitosan–pectin hydrogels cross-linked with β-glycerophosphate [13]. Articular canine chondrocytes were shown to sense their intrinsic mechanical environment. After 1 week on non-cross-linked and cross-linked chitosan films, the cells possessed a flattened and spread morphology, with cell proliferation appearing to be higher on more rigid cross-linked films [16]. In comparison with proteins, polysaccharide matrices can provide a very broad range of stiffness, which can be achieved by different chemical and physical cross-linking strategies.

Although high stiffness promotes adherent cell proliferation in 2D culture, the development of 3D cancer models is challenging, since a high cross-linking degree or high polymer concentration in stiff hydrogels result in spatial constraints and low permeability for nutrients that negatively affect the size, morphology and density of the growing spheroids [13,17]. Therefore, the fabrication of macroporous scaffolds is promising for providing simultaneously required scaffold stiffness, efficient transport of nutrients and cell metabolites, and sufficient inner space for 3D cell growth [2,17,18,19,20]. Among different methods of porous materials fabrication, including salt/particle leaching, freeze drying, phase separation, and gas foaming, cryogelation, i.e., the polymerization of monomers or cross-linking of polymers at subzero temperature with the subsequent removal of porogenic ice crystals by thawing, is a simple, green and efficient approach. 

We have recently shown that biocompatible cryogels with Young′s moduli up to 90 kPa can be obtained via chitosan cross-linking with diglycidyl ethers of ethylene glycol and poly(ethylene) glycol [21] that makes such supermacroporous materials promising for culturing adhesive CRC cells and developing 3D tumor models. Taking into account the even higher potential of a commercially available chitosan derivative, carboxymethyl chitosan (CMC), for application in tissue engineering [18,22], we aimed here to develop fabrication methods of chitosan- and CMC-based macroporous scaffolds cross-linked with 1,4-butandiol diglycidyl ether and study the influence of surface chemistry, morphology, and stiffness of these matrices on HCT 116 cell growth. 

## 2. Materials and Methods

### 2.1. Materials

Low molecular weight (30 kDa) chitosan and N,O-(carboxymethyl)chitosan (CMC) were purchased from BioLog Heppe GmbH (Landsberg, Germany). The chitosan degree of acetylation (DA = 0.9) and CMC degree of carboxyalkyl substitution (DS = 1.49) and monomer composition were determined by ^1^H NMR spectroscopy (Figure S1 and Table S1, Supplementary Materials). Cross-linking agent—1,4-butanediol diglycidyl ether (BDDGE) was purchased from Sigma-Aldrich (St. Louis, MO, USA).

### 2.2. Fabrication of Cryogels

The 3%-chitosan solution was prepared by dissolution of the polymer powder in hydrochloric acid at an equimolar NH_2_:HCl ratio; then, pH was adjusted to 5 with 0.1 M NaOH solution. CMC solution of the same concentration was prepared in distilled water, and the resulting pH of the solution was 10.2. The calculated amounts of the cross-linker (BDDGE), corresponding to the molar ratios polymer (BDDGE 2:1, 1:1, 1:2, and 1:4; see Table S2, Supplementary Materials for an example) were added dropwise under constant stirring to the polymer solutions. Then, solutions were immediately placed into the plastic syringes and kept in a freezer (Liebherr, Kirchdorf an der Iller, Germany) at –10 °C during 12 or 7 days for chitosan and CMC, respectively. After thawing, the cryogels were washed with distilled water using a peristaltic pump (Ismatec, Wertheim, Germany) to remove unreacted chemicals.

### 2.3. Characterization of Hydrogels

#### 2.3.1. Elemental Analysis 

CHN contents in the reaction products (cryogels) were determined in triplicates using a EuroEA3000 CHNS analyzer (Eurovector, Pavia, Italy), and the degree of modification (DM) was calculated using the following formula:

DM=CNcryogel−CNpolymer10

where C/N_cryogel_ and C/N_polymer_ are the atomic carbon/nitrogen ratios in cryogels and polymer precursors (chitosan and CMC), respectively; 10 is the number of carbon atoms in cross-linker BDDGE. 

#### 2.3.2. Rheological Properties and Swelling

The rheological properties of the hydrogels were investigated by recording frequency sweeps in the range between 1 and 100 Hz at a temperature of 25 °C and a constant strain of 5% (which was within the linear viscoelastic region) using a Physica MCR 301 rheometer (Anton Paar GmbH, Graz, Austria) with a plate–plate measuring system with a diameter of 25 mm.

The Young′s modulus (E) was calculated from the linear region of the penetration curve obtained by pressing down cylindrically shaped cryogel with a diameter of 25 mm and height of 7–8 mm using a measuring plate at a constant speed of 0.01 mm/s and measuring normal force (F_N_) and gap with a Physica MCR 301 rheometer:

E=l0·FNS·Δl

where F_N_ is the normal force, l_0_ is the initial sample height, Δl is the change in the sample height, and S is the area of the material.

Swelling of the cryogels was determined from the difference in weights of swollen and dry material (the measurements were performed for freshly prepared cryogels from wet to dry state). To determine the contribution of water absorbed in macropores to the total swelling, the swollen cryogels were first squeezed by fingers and weighted. 

Morphology of the cryogels was analyzed using confocal laser scanning microscopy (CLSM), as described in Section 2.3.6.

#### 2.3.3. Cell Cultivation

The 24-well culture plates (TPP, Trasadingen, Switzerland), ultra-low attachment plates (Corning^®^ Costar^®^, Somerville, MA, USA) and cryogel disks were used to grow HCT 116 cells (Sigma-Aldrich, St. Louis, MO, USA) in adhesive, suspension and 3D conditions, consequently.

HCT 116 cells were seeded in adhesive and ultra-low attachment plates at a density 100 × 10^3^ cells/well in 1 mL of Dulbecco’s modified Eagle’s medium (DMEM, #12800017, Gibco™, Thermo Fisher Scientific, Altrincham, UK) supplemented with 10% (*v*/*v*) fetal bovine serum (FBS, HyClone, Logan, UT, USA), 3.7 mg/mL sodium bicarbonate (Sigma-Aldrich), 1× mixture of non-essential amino acids (MEM NEAA, Waltham, MA, Gibco), 100 U/mL penicillin (Gibco), and 100 µg/mL streptomycin (Gibco).

The fabricated cryogel was cut to disks (diameter of 8 mm, thickness of 4 mm), and each disk was placed in well of a 24-well TPP culture plate, which was consistently washed with 5 mL of Dulbecco’s phosphate buffer saline (DPBS, Sigma-Aldrich) without Ca^2+^ and Mg^2+^, and 5 mL of DMEM with additives. All liquid was squeezed from the cryogel. The HCT 116 cells were seeded on the top of a cryogel disk at a density 100 × 10^3^ cells/cryogel in 1 mL of DMEM with additives.

All samples were cultivated at +37 °C, 5% CO_2_ and 90% relative humidity. The medium has been changed each 2 days. The cells were monitored daily under a CKX41 inverted microscope (Olympus, Shinjuku City, Tokyo, Japan) equipped with phase-contrast optics and imaged with an Axiocam 105 color digital camera (Carl Zeiss, Oberkochen, Germany) in ZEN 2 (blue edition, Carl Zeiss). The viability and functional activity of cells were analyzed after 1, 2, 3, 7, and 14 days of cultivation in cryogels.

To analyze the cytotoxicity of the cross-linker, the BDDGE solution was added to HCT 116 cells cultivated in adhesive 24-well TPP plates at concentrations corresponding to BDDGE:chitosan cross-linking ratios 1:1 (62 mg/mL), 1:2 (31 mg/mL), 1:4 (15.5 mg/mL), and 1:8 (7.75 mg/mL). After incubation for 3, 24, and 48 h, the cells were analyzed by microscopy and flow cytometry, as described in Section 2.3.5.

#### 2.3.4. Permeability of Cryogels

The 2 mL cryogel in a 2.5 mL syringe was washed with 10 mL of DPBS and 5 mL of DMEM with additives. The HCT 116 cells were seeded on a top of cryogel at a density 5 × 10^5^ cells/cryogel in 1 mL of DMEM. The cell suspension was allowed to penetrate into the gel. The syringe was placed on a 5 mL centrifuge tube (Axygen, Union City, CA, USA); and 2 mL of DMEM was added on top of the cryogel. Cells eluted with the medium under the gravity force were collected in the centrifuge tube. The syringe was transferred on a new centrifuge tube, and the washing procedure has been repeated until 18 mL of medium was fed through the gel. 

#### 2.3.5. Cell Staining and Flow Cytometry

All collection tubes with HCT 116 cells washed from cryogels were centrifuged at 500× *g* for 5 min. The pellets of cells were stained with 100 µL of 1 µM SYTO 9 (Invitrogen, Waltham, MA, USA) in the dark at room temperature for 10 min and then diluted with 100 µL of DPBS before flow cytometry.

The cells were cultivated in adhesive, ultra-low attachment plates, and cryogels were washed with 1 mL of DPBS, detached with the solution of 0.05% (*w*/*v*) trypsin—0.02% (*w*/*v*) EDTA from the wells and each other, and centrifuged at 500× *g* for 5 min. A pellet of trypsinized cells from a single well of a 24-well plate or cryogel disk was re-suspended in 100 µL of DPBS with 10 µM 2’,7’-dichlorodihydrofluorescein diacetate (H_2_DCFDA) (Sigma-Aldrich) to assess the mitochondrial activity, 1 µM TO-PRO-3™ (Invitrogen) to detect apoptotic cells, and 1 µg/mL DAPI (GERBU Biotechnik GmbH, Heidelberg, Germany) to stain dead cells. The cell suspension was incubated in the dark at room temperature for 10 min and then diluted with 150 µL of DPBS before flow cytometry. The detailed description of flow cytometrical analysis is given in [23]. 

#### 2.3.6. Confocal Laser Scanning Microscopy (CLSM)

Morphology of the swollen (never dried) chitosan cryogels stained with fluorescein and CMC cryogels stained with rhodamine was investigated using a Carl Zeiss LSM 800 confocal laser scanning microscope (Germany) with regular 10× and 20× objective lens. The excitation and emission wavelengths used were set at 488 and 530 nm for fluorescein and at 561 and 572 nm for rhodamine, respectively. Images were generated by optical sectioning in the xy-planes along the z-axis with 30 optical sections with 2.5 μm intervals. The optical-section series were projected as single images and exported as TIFF files. The pore size distributions were calculated using the ImageJ software [24]. 

Cryogel disks with cultured cells were fixed in 4% paraformaldehyde (PFA; Sigma) in phosphate buffer (PBS), pH 7.8, for 30 min at +4 °C and rinsed three times with cold PBS. To detect filamentous actin, gels with cells were incubated overnight in a solution of tetramethylrhodamine-conjugated phalloidin (Santa Cruz Biotechnology, Dallas, TX, USA) at +4 °C; then, they were washed three times with cold PBS. Then, the samples were stained with 10 μg/mL 4′,6′-diamidino-2-phenylindole (DAPI, Sigma-Aldrich) in PBS to reveal the nuclei. The stained material was stored in PBS with added preservative (ProClin, Sigma-Aldrich) in the dark at +4 °C. Immediately before the microscopy analysis, the gel section was placed in Vectashield medium in a Petri dish with a thin (0.17 mm) glass bottom. For each sample, several fields were scanned at a magnification 10× and at a magnification 20× to visualize the interaction of cells with the matrix. Z-projections and multichannel images were assembled using the ImageJ software (NIH, USA) [24].

## 3. Results and Discussion

### 3.1. Fabrication of Cryogels via Chitosan and CMC Cross-Linking with BDDGE 

Diglycidyl ethers of glycols (DGE) are commonly used in biomedicine for scaffolds and fillers fabrication to control their stiffness and in-body enzymatic degradation rate [25,26], although the cytotoxicity of this type of cross-linkers is still an issue [25,27]. Cross-linking with DGE can be performed via amino-, hydroxyl-, and carboxyl groups [28] that covers most important classes of polymers used as matrices in cell culturing and tissue engineering [20,26,29,30]. However, efficient cross-linking with DGE occurs only at pH > 10 [27,31], when chitosan is not soluble. Under alkaline conditions, due to the low reactivity of chitosan and CMC, cross-linking proceeded only at elevated temperature: 45 °C for chitosan with 1,4-butandiol DGE (BDDGE) [16] and 60 °C for CMC with poly(ethylene glycol) (PEGDGE) [29]. In addition, BDDGE and PEGDGE were used at concentrations (above 0.5%) to yield hydrogels [16,29]. This may complicate experiments with live cells, because 100–1000 ppm of BDDGE was shown to significantly decrease the viability of keratinocytes and fibroblasts [25].

**Figure 1:**
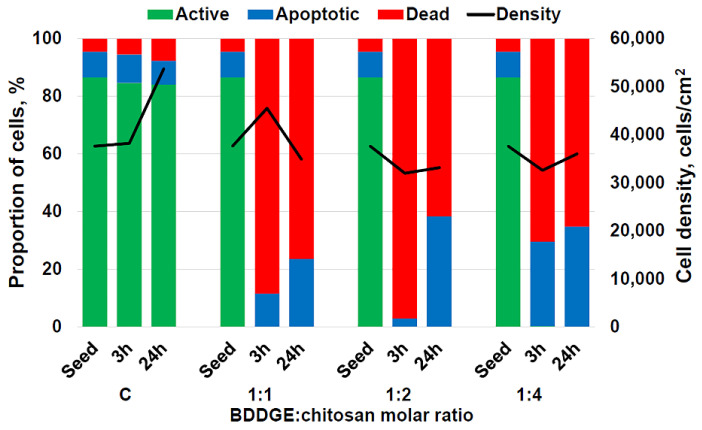
The results of flow cytometrical analysis of control (C) human colon carcinoma cells (HCT 116) and cells cultivated for 3 and 24 h in the presence of BDDGE at concentrations corresponding to a BDDGE:chitosan molar ratio (in 3% polymer solution) of 1:1, 1:2, and 1:4 (see example of calculations in Table S2, Supplementary Information). The cells were stained with H_2_DCFDA to assess the mitochondrial activity, TO-PRO-3™ to detect apoptotic cells, and DAPI to stain dead cells. The data are presented as a mean of three independent experiments. Standard deviations did not exceed 5%.

Taking into account conditions of chitosan cross-linking with BDDGE [16], and ethylene glycol DGE (EGDGE) and PEGDGE [21], before the fabrication of scaffolds for the development of 3D tumor models, we have estimated BDDGE cytotoxicity for HCT 116 cells at concentrations corresponding to BDDGE:chitosan molar ratios of 1:1, 1:2, and 1:4. Figure 1 demonstrates the high toxicity of the cross-linker: even 3 h incubation with the lowest BDDGE:chitosan ratio of 1:4 induced more than 70% cell death and 30% apoptosis; moreover, the cell proliferation was suppressed in the presence of BDDGE. 

The evolution of rheological properties of polymer solutions after the addition of BDDGE at a molar ratio to polymer of 1:4 (Figure S2, Supplementary Materials) shows that loss moduli (G″) were higher than storage moduli (G′) after 24 h, indicating that gels were not formed neither by chitosan nor by CMC. Since no active cells were observed after 24 h incubation of HCT 116 cells with BDDGE at all tested concentrations, BDDGE cannot be applied for the direct encapsulation of cells in hydrogels. The cells should be seeded in BDDGE cross-linked scaffolds only when cross-linking is completed. 

**Scheme 1:**
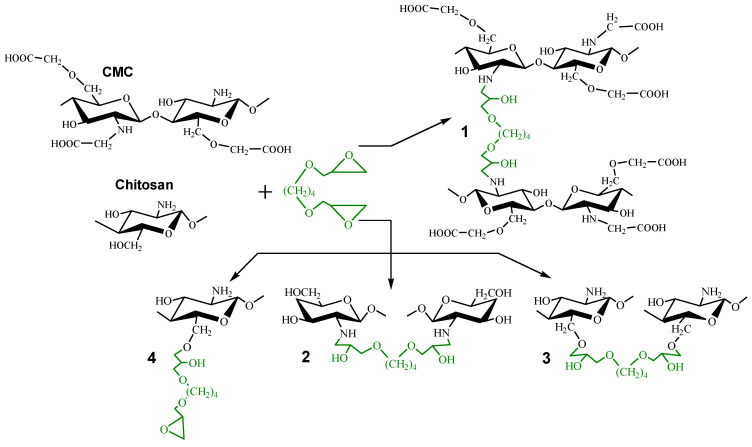
Scheme of interaction of chitosan and CMC with BDDGE.

Rheological data show that despite the alkaline medium in CMC solution being more favorable for cross-linking, BDDGE was a more efficient cross-linker for chitosan (Figure S2, Supplementary Materials). At 72 h after BDDGE addition, the G′/G″ ratio for chitosan hydrogel reached a value of 7.7, when gelation has not yet started in CMC solution (G″ > G′). In contrast, 7 days after the addition of BDDGE, the difference in storage moduli of CMC (44 Pa) and chitosan (G′ = 6.7 kPa) hydrogels was still significant. Since it was shown that BDDGE in alkaline media reacts preferentially with hydroxyl groups of hyaluronic acid [31], low CMC reactivity can be related to its high degree of O-carboxyalkyl substitution (DS = 1.20). So, according to the monomer composition of CMC (Table S1, Supplementary Materials), cross-linking is possible via free amino groups in 46% of monomer units (Scheme 1, structure 1) and less reactive unsubstituted hydroxyl groups. 

Cross-linking at subzero temperature reduces reactivity, but at the same time, it increases the probability of cross-link formation due to the cryo-concentration effect, i.e., decrease in the critical polymer concentration required for gelation [32]. In preliminary experiments, we observed that the temperature (−10 °C) used for chitosan cross-linking with EGDGE and PEGDGE [21] was optimal for chitosan and CMC cross-linking with BDDGE. At lower temperature (−15 °C and −20 °C), the loss of reactivity did not result in cross-linking at an equimolar BDDGE:polymer ratio during 14 days. Another reason for cryogel fabrication at higher temperature is the observation that a lower cryogelation temperature leads to the smaller pore size [20,26] that is not desirable for scaffolds for 3D tumor models development. Moreover, the fabrication of macroporous scaffolds from CMC hydrogels reported earlier was possible only via freeze-drying. The irregular comb-like porous structure of this material seems unsuitable for 3D cell culturing [29].

Cryogels of chitosan and CMC cross-linked at BDDGE:polymer ratios from 2:1 to 1:4 were formed at a temperature of −10 °C after 7 and 3 days, respectively. However, the prolonged cross-linking time was shown earlier to yield more round-shaped pores with more homogeneous size distribution and thinner polymer walls [26]. Therefore, we have decided to increase the cross-linking time to 7 days for CMC and 12 days for chitosan to assure the completeness of the reaction and formation of regular porous structure. 

**Figure 2:**
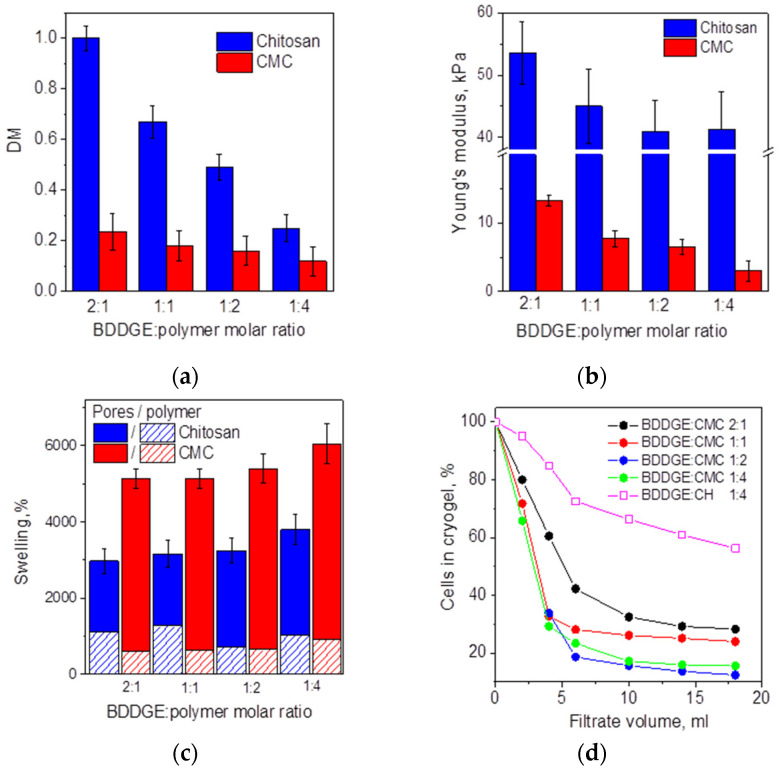
Dependencies of polymer modification degrees with cross-linker (**a**), Young′s moduli (**b**) and swelling (**c**) on BDDGE:polymer molar ratios used for the fabrication of carboxymethyl chitosan (CMC) and chitosan cryogels. Permeability of cryogels for HCT 116 cells (percentage of cells remained in 2 mL monolith cryogels after elution with DMEM) (**d**).

Figure 2 shows the comparative characteristics of chitosan- and CMC-based cryogels. Although the degree of polymer modification (DM) with BDDGE cannot be attributed to cross-linking density due to the possible formation of grafts (structure 4, Scheme 1), it confirms the lower reactivity of CMC in comparison with chitosan. Since the DM of CMC was below 0.25 even at the highest BDDGE:polymer molar ratio and only slightly decreases with a decrease in BDDGE concentration, we have concluded that a high carboxyalkyl substitution of CMC indeed limited the number of reactive functional groups, which could participate in the cross-linking reaction. Chitosan can react with BDDGE via abundant hydroxyl and amino groups (Scheme 1, structures 2 and 3), so the DM values were close to the theoretically expected (Figure 2a). A decrease in crosslinking density resulted in a decrease in Young′s modulus (Figure 2b) and increase in swelling for both series of cryogels (Figure 2c). 

**Figure 3:**
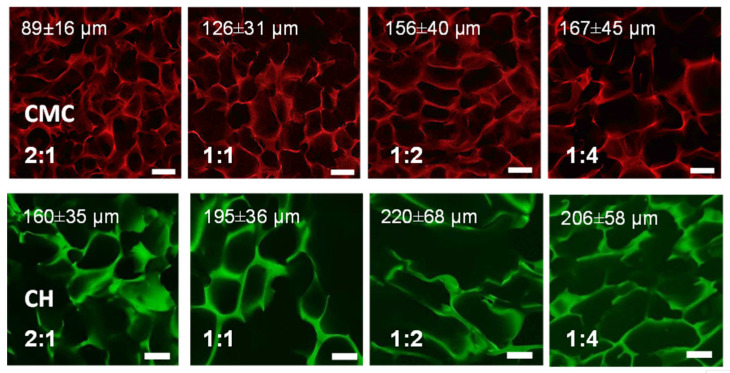
Confocal laser scanning microscopy (CLSM) images and average pore sizes of CMC- and chitosan (CH)-based cryogels fabricated at different BDDGE:polymer molar ratios. Scale bar—100 µm.

All cryogels had high equilibrium swelling; however, due to the much lower DM of CMC, the contribution of the free-flowing water (macropores) to the total swelling was notably higher for CMC cryogels (Figure 2c). These differences in properties of two cryogel series were in good agreement with their morphology (Figure 3). CMC cryogels had significantly thinner pore walls and more homogeneous pore size distribution with the average pore size increasing with decreasing BDDGE:polymer ratios. All CMC cryogels supported a flow-through regime and were highly permeable for HCT 116 cells (Figure 2d). Due to the much thicker pore walls and lower porosity of the highly cross-linked chitosan cryogels with high DM, only cryogel obtained at a BDDGE:chitosan ratio of 1:4 showed flow-through properties. However, despite the large pore size in comparison with CMC cryogel obtained under the same cross-linking conditions, chitosan cryogel was less permeable for cells, which was probably due to its higher rigidity. It shall be mentioned that for the sake of comparison of cryogels obtained under the same cross-linking conditions, we do not report here chitosan cryogels with lower cross-linking density, since BDDGE:CMC 1:4 was the lowest ratio at which CMC cryogel could be formed. 

Taking into account the importance of a sufficient transport of nutrients and cell metabolites, for the further experiments on HCT 116 cell culturing in the developed sponge-like macroporous scaffolds, we have used a complete series of CMC cryogels with Young′s moduli in the range 3–13 kPa and chitosan cryogel with Young′s modulus (41 kPa), since chitosan cryogels with a higher cross-linking density were impermeable.

### 3.2. 3D Culturing of HCT 116 Cells 

**Figure 4:**
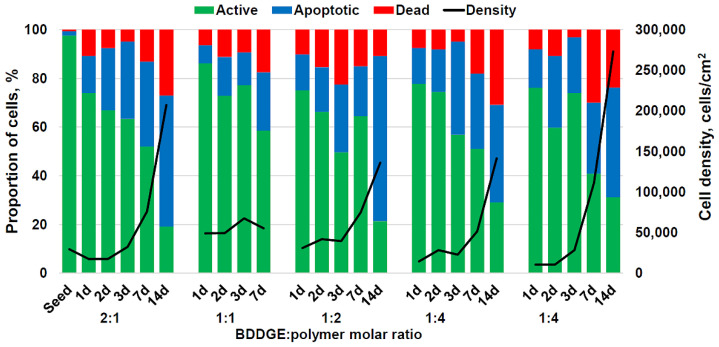
The results of flow cytometrical analysis of human colon carcinoma cells (HCT 116) cultivated for 1, 2, 3, 7 and 14 days in CMC cryogels (1–4) and chitosan cryogel (5) cross-linked at various BDDGE:polymer molar ratios. The cells were stained with H_2_DCFDA to assess the mitochondrial activity, TO-PRO-3™ to detect apoptotic cells, and DAPI to stain dead cells. The data are presented as a mean of three independent experiments. Standard deviations did not exceed 5%.

Evaluation of the CMC cryogels cytotoxicity for HCT 116 cells confirmed that more than 50% of cells remained active during 7 days of cultivation regardless of the cross-linking density (Figure 4). Cell viability in chitosan cryogel was comparable with that in CMC cryogels at the first days of culturing but dropped on the 7th day with a drastic increase in cell density that was not observed for CMC cryogels. 

**Figure 5:**
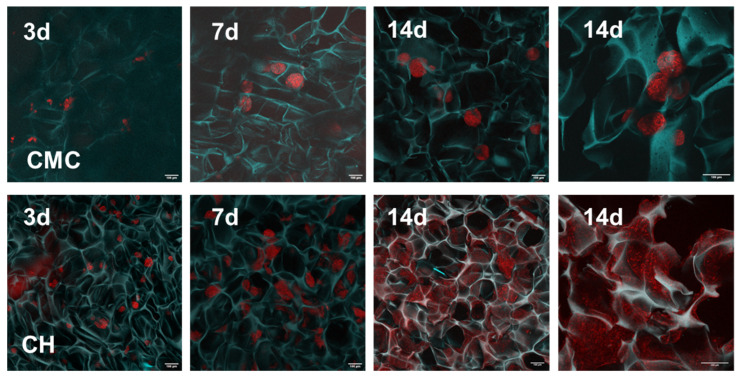
Confocal laser scanning microscopy (CLSM) images of CMC- and chitosan (CH)-based cryogels (BDDGE:polymer ratio of 1:4) with HCT 116 cells after 3, 7, and 14 days of cultivation. Scale bar—100 µm.

Monitoring HCT 116 cells growth using CLSM revealed a significant difference in the morphology of 3D cell aggregates in CMC and chitosan cryogels (Figure 5). After three days in culture, we observed small loose cell clusters with 11.5 ± 6 cells in BDDGE:CMC cryogel at a 1:4 ratio and 17.6 ± 7.1 cells in BDDGE:chitosan cryogel at a 1:4 ratio. After seven days in culture, round-shaped tight HCT 116 spheroids with average size of 104 ± 30 µm and 75 ± 10 µm were observed in cryogels cross-linked at BDDGE:CMC ratios of 1:4 and 1:2, respectively. Interestingly, the stiffest CMC cryogel obtained at a BDDGE:CMC of 2:1 ratio promoted cell adhesion and fast growth in the first 3 days, but the average size of spheroids was limited to 80 ± 36 µm, and they were more heterogeneous than in other cryogels. CLSM images for BDDGE:CMC of 1:2 and 2:1 cryogels are shown in the Supplementary Materials, Figure S3.

In cryogels with a Young′s moduli of 3–13 kPa, the adhesion of HCT 116 cells to each other prevailed over the adhesion to the surface. The rate of HCT 116 spheroid growth in CMC cryogels was significantly higher, while the size distribution was more narrow, in comparison with most of the earlier reported hydrogels, e.g., [1,13,33]. Due to the confinement imposed by the porous structure and, possibly, due to the limited access of cells inside spheroids to nutrients, the spheroid size did not increase between the 7th and 14th days of cultivation in CMC cryogels. The plateau in HCT 116 spheroids size reached by the 10–14th day of culturing was observed previously [13,15,34].

HCT 116 cells in stiff chitosan cryogel (Young′s modulus 41 kPa, pore size 206 ± 58 µm) were preferentially attached to the surface of the pore walls and formed after 3 days in culture numerous tightly packed clusters with the average size of 44 ± 12 µm. After 7 days of cultivation, the cells occupied a large surface area, repeating the shape of the pore walls and forming epithelial-like structures (Figure 5). In contrast to CMC cryogel, HCT 116 cells growth in chitosan cryogel was not limited by the pore size; cells migrated in interconnected porous structure forming by 14th day continuous sheets with thickness > 150 µm that correlates with the exponential growth of cell density observed for this cryogel from cytometrical analysis (Figure 4). The formation of sheet-like 3D structures by CRC cells was very rarely observed in vitro; however, a recent comparison of HT 29 and HCT 116 cell growth in a biomimetic model including a complex, vascularized stroma showed that more invasive HCT 116 grew as thin sheets with frayed edges, while less invasive HT 29 formed round intact clusters [35]. Since the higher values of elastic modulus of CRC tissue correlated with a larger tumor size, venous invasion, perineural invasion, poorly differentiated clusters and elastic laminal invasions [12], the developed rigid chitosan cryogels can provide a promising alternative to soft hydrogels lacking cell–matrix interactions and promoting only the formation of spheroids. 

It shall be emphasized that a difference in the chemical structure of chitosan and CMC cannot explain the drastic difference in morphology of HCT 116 micro-tumors formed in two types of cryogels. Only a few spheroids with an average diameter of about 150–200 μm were shown to form after 2 weeks in chitosan hydrogel cross-linked with beta-glycerophosphate [13]. 

**Figure 6:**
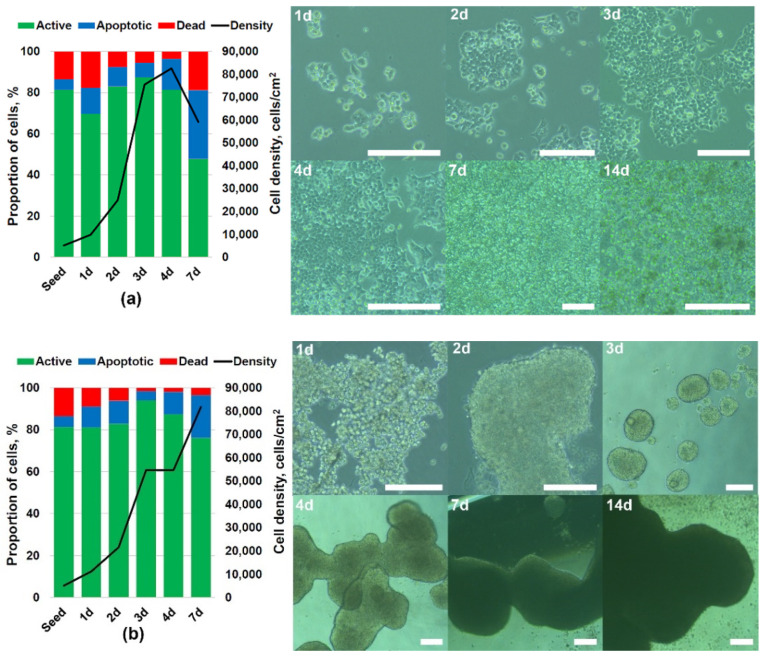
The results of flow cytometrical analysis and microscopic observation of human colon carcinoma cells (HCT 116) cultivated for 1, 2, 3, 4, and 7 days in adhesive (**a**) or ultra-low attachment (**b**) plates. The cells were stained with H_2_DCFDA to assess the mitochondrial activity, TO-PRO-3™ to detect apoptotic cells, and DAPI to stain dead cells. The data are presented as a mean of three independent experiments. Standard deviations did not exceed 5%. The cells were imaged under a CKX41 inverted microscope (Olympus, Shinjuku City, Tokyo, Japan) equipped with phase-contrast optics. Scale bar—200 µm.

There was an explicit similarity between HCT 116 micro-tumor morphology in soft (CMC cryogel) or stiff (chitosan cryogel) and in ultra-low attachment or adhesive plates. An attachment of cells on the polystyrene culturing plates was observed already after several hours after seeding. The next day, cells spread and started to proliferate (Figure 6a). The cells grew exponentially until the 4th day, when almost all the available surface was covered by cells. They continued to grow, forming several cell layers. Overgrowth and contact inhibition decreased cellular activity and cell density and increased apoptosis. In the case of the cryogel disk, there were much more surface area and volume available for the cell growth. Cells continued to grow, covering all outer sides of the cryogel disk and inner pores surfaces. Still, cells located in deeper layers were probably suppressed by the lack of nutrients and oxygen starvation. In methylcellulose–hyaluronic hydrogel, HCT 116 cells after 7 days in culture grew as spheroids with a population of dead cells in the core [15] that is typical for tumors. In chitosan cryogel, the size of elongated 3D structures formed by the 7th day of culturing corresponded to three to four layers of cells and the number of apoptotic cells also increased; however, by the 14th day in culture, the cell density continued exponential growth that allows using such micro-tumor models in long-time experiments. 

The cultivation of HCT 116 cells in ultra-low attachment plates resulted in an aggregation of cells to each other. The lack of the cell contacts with a polystyrene surface did not stop cell proliferation. Round-shape spheroids with thousands of cells in each were formed on the 3rd day in culture (Figure 6b). These spheroids then continued to aggregate into large cell conglomerates with millions of cells in each. The growth was limited by the availability of nutrients from the medium to cells in the inner part of conglomerates and decreased after prolonged cultivation (the data not shown). Similar behavior was observed in Matrigel and methylcellulose–hyaluronic hydrogel with stiffness of 0.4–0.6 kPa, when fast HCT 116 spheroids growth was observed in the first days and followed by a decrease due to the limitation of nutrition and oxygen supply and/or accumulation of cell metabolic waste [15,36]. The advantage of CMC cryogels over ultra-low attachment plates is the possibility to control micro-tumor growth by the limited inner space of pores, and long-term stability of spheroids due to sufficient transport of nutrients and metabolites to cells.

## 4. Conclusions

The method for the fabrication of sponge-like supermacroporous scaffolds (cryogels) by cross-linking low molecular weight chitosan and carboxymethyl chitosan (CMC) with 1,4-butandiol diglycidyl ether (BDDGE) at −10 °C and BDDGE:polymer molar ratios from 2:1 to 1:4 have been developed. The morphology, chemical composition, rheological properties, stiffness and swelling of the cryogels have been studied using confocal laser scanning microscopy (CLSM), elemental analysis, uniaxial compression and gravimetry. 

It was shown that due to the lower degree of modification of CMC with a cross-linking agent, CMC cryogels had significantly thinner pore walls and a more homogeneous pore size distribution with an average pore size increasing from 89 to 167 µm at decreasing BDDGE:CMC ratio. All CMC cryogels demonstrated high swelling (5000–6000%), good permeability for HCT 116 cells and relatively low stiffness with Young′s moduli in the range from 3 to 13 kPa. Only one chitosan-based cryogel (BDDGE:chitosan ratio of 1:4, Young′s modulus 41 kPa) supported flow-through regime and was selected for 3D cell culturing. Although BDDGE showed high cytotoxicity, cross-linked cryogels did not show a negative effect on HCT 116 cells growth. 

For the first time, we have demonstrated that the morphology of a colorectal cancer (CRC) micro-tumor can be controlled by selection of the polymer for the culturing scaffold fabrication. CMC cryogels have shown non-adhesive properties and stimulated the formation of HCT 116 spheroids due to the lack of cell–matrix interactions, while stiff chitosan cryogels supported the cell–matrix interaction and guided HCT 116 growth as epithelia-like continuous sheets. Taking into account earlier reported correlations between the stiffness of a CRC tumor environment and cell invasiveness, the designed scaffolds can be of interest for drug screening in oncology and further developments in 3D culturing of adherent cancer cell lines.

## Data Availability

Data available from the authors upon request.

## Supplementary Materials

The following are available online at https://www.mdpi.com/article/10.3390/biomimetics7020056/s1, Table S1. Monomer composition of carboxymethyl chitosan (CMC). Table S2. Calculations of reagents quantities for chitosan cryogels fabrication. Figure S1: The 400 MHz ^1^H NMR spectra of N,O-(carboxymethyl)chitosan. Figure S2. Evolution of mechanical spectra of 3% CMC (a) and chitosan (b) solutions after addition of cross-linker BDDGE: filled symbols—storage modulus (G′), open symbols—loss modulus (G″), half-filled—complex viscosity. Figure S3. Confocal laser scanning microscopy (CLSM) images of CMC- cryogels with HCT 116 cells after 7 days (a), and 14 days (b) of cultivation (BDDGE:polymer ratio of 1:2) and after 3 days (c), and 7 days (d) of cultivation (BDDGE:polymer ratio of 2:1), scale bar 100 µm.
