# Supplementary material for: Sponge-like Scaffolds for Colorectal Cancer 3D Models: Substrate-Driven Difference in Micro-Tumors Morphology
Source: Biomimetics (Basel). 2022 May 5;7(2):56. doi: 10.3390/biomimetics7020056 (PMC9149916; doi:10.3390/biomimetics7020056)
Supplement: Supplementary file 1 [file biomimetics-07-00056-s001.zip › biomimetics-1689905-supplementary.pdf]

Supplementary materials

Sponge-like scaffolds for colorectal cancer 3D models: substrate driven difference in micro tumors morphology

Andrey Boroda [1], Yuliya Privar [2], Mariya Maiorova [1], Anna Skatova [2], and Svetlana Bratskaya [2,*]

[1] A.V. Zhirmunsky National Scientific Center of Marine Biology, Far Eastern Branch of Russian Academy of Sciences, 17, Palchevskogo street, 690041 Vladivostok, Russia; borodandy@gmail.com

[2] Institute of Chemistry Far Eastern Branch of the Russian Academy of Sciences, 159, prosp.100-letiya Vladivostoka, 690022 Vladivostok, Russia; sbratska@ich.dvo.ru

* Correspondence: sbratska@ich.dvo.ru (S.B.)

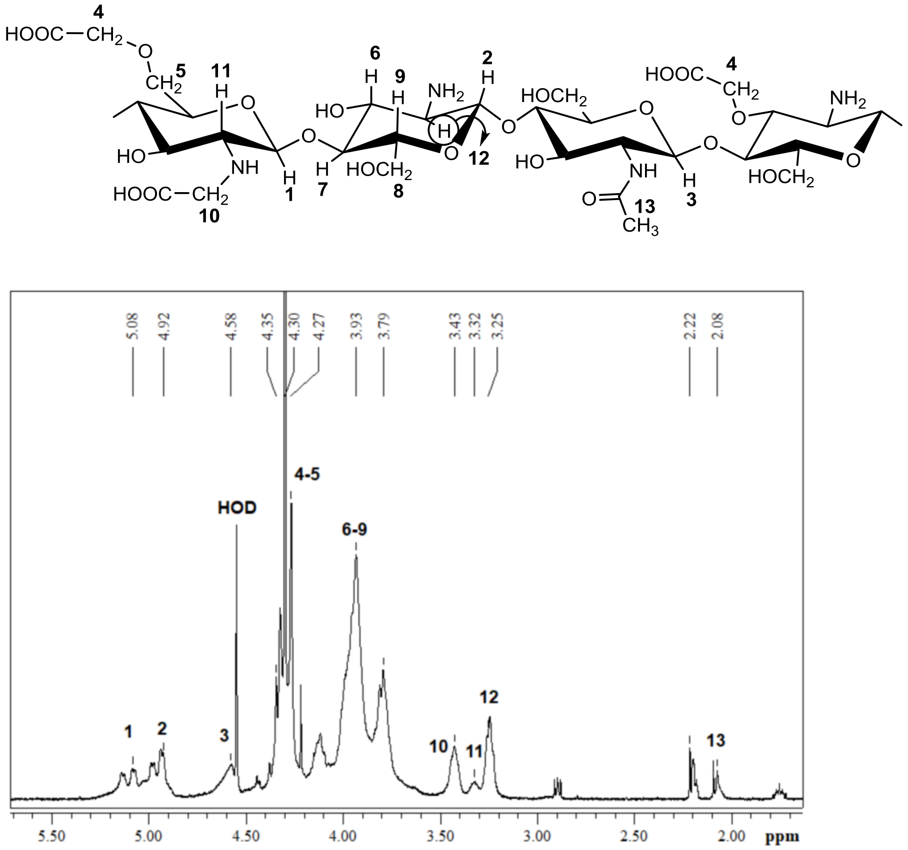

Figure S1. 400 MHz ^{1}H NMR spectra of N,O-(carboxymethyl)chitosan

Table S1. Monomer composition of carboxymethyl chitosan (CMC)

DA[1]	DS_{tot}[2]	N-DS[3]	O-DS[4]	Monomer composition			
				NH_2	NHR	NR_2	$NHCOCH_3$
0.25	1.49	0.29	1.20	0.46	0.29	0	0.25

[1] - Degree of acetylation, [2] - Degree of carboxyalkyl substitution (total), [3] - Degree of N-carboxyalkyl substitution, [4] - Degree of O-carboxyalkyl substitution

Table S2. Calculations of reagents quantities for chitosan cryogels fabrication

Chitosan molecular weight - 163 g/mol

BDDGE molecular weight - 202 g/mol

BDDGE:chitosan molar ratio	3% chitosan solution, g	60% BDDGE solution, g
2:1	5	0.62
1:1	5	0.31
1:2	5	0.155
1:4	5	0.0775

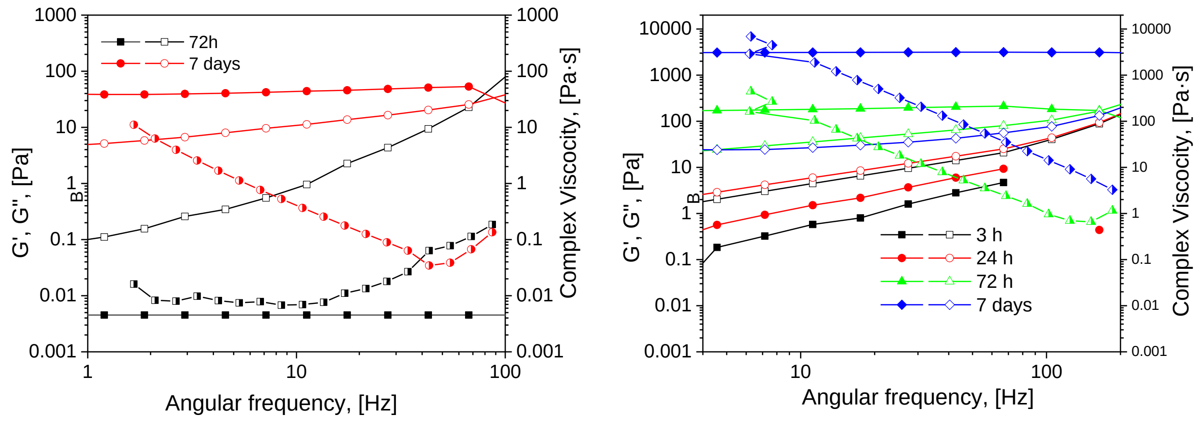

(**a**) (**b**)

Figure S2. Evolution of mechanical spectra of 3% CMC (a) and chitosan (b) solutions after addition of cross-linker BDDGE: filled symbols – storage modulus (G'), open symbols – loss modulus (G''), half-filled – complex viscosity storage modulus (G'), open symbols – loss modulus (G''), half-filled – complex viscosity

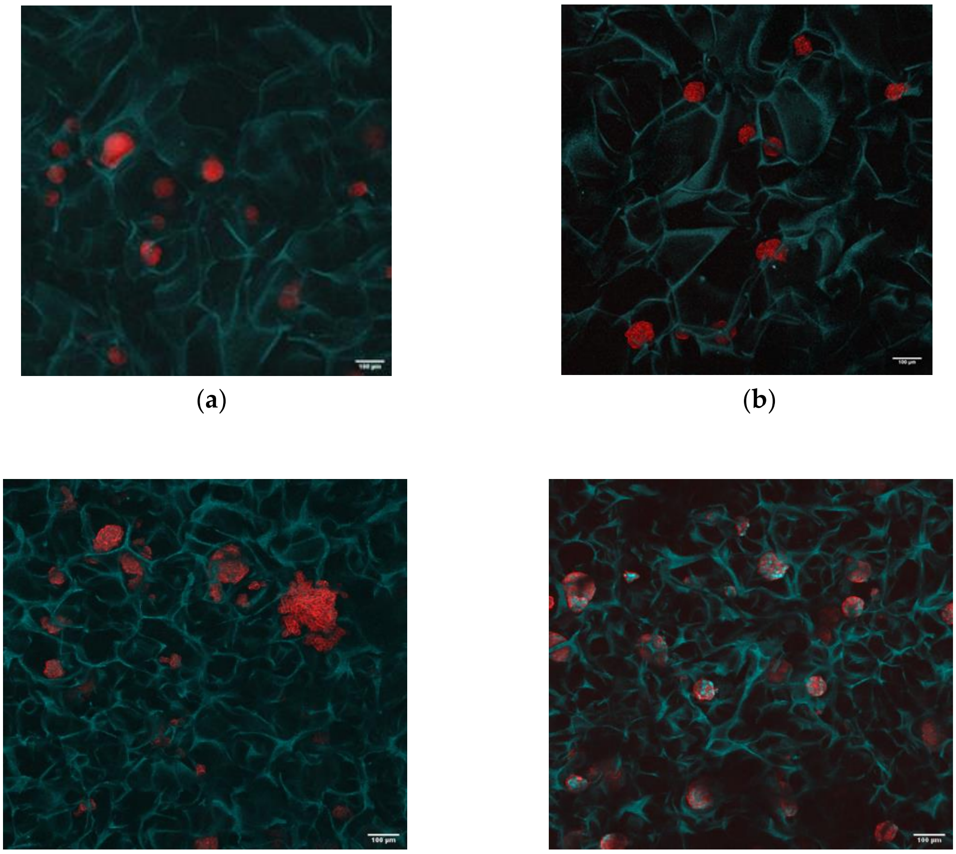

(**a**) (**b**)

(**c**) (**d**)

Figure S3. Confocal laser scanning microscopy (CLSM) images of CMC- cryogels with HCT 116 cells after 7 days (a), and 14 days (b) of cultivation (BDDGE:polymer ratio of 1:2) and after 3 days (c), and 7 days (d) of cultivation (BDDGE:polymer ratio of 2:1), scale bar – 100 µm.
